# Retrospective analysis of surgical outcomes and prognosis in elderly patients with gynecologic cancers

**DOI:** 10.1097/MD.0000000000044657

**Published:** 2025-10-03

**Authors:** Zhengyi Chen, Yuanqing Chen

**Affiliations:** a Department of Obstetrics and Gynecology, The First Affiliated Hospital of Xiamen University, Xiamen, Fujian Province, China.

**Keywords:** complications, gynecologic malignancy, logistic regression analysis, postoperative recovery, survival rate

## Abstract

With the progressive aging of the population, the number of elderly patients with gynecologic malignancies is steadily increasing. Due to the presence of multiple comorbidities and diminished postoperative recovery capacity, this population is associated with considerable variability in surgical outcomes, highlighting the urgent need for optimized perioperative management. This study aimed to investigate key factors influencing postoperative recovery quality and 1-year prognosis in elderly patients with gynecologic cancers, in order to provide evidence for individualized clinical management. A total of 132 elderly patients (aged ≥ 65 years) who underwent surgical treatment for gynecologic malignancies at our hospital between January 2023 and May 2024 were retrospectively enrolled. Based on postoperative recovery quality and recurrence/survival status within 1 year, patients were categorized into a good prognosis group (n = 68) and a poor prognosis group (n = 64). Baseline characteristics, intraoperative parameters, postoperative recovery indicators, and long-term outcomes were compared between the 2 groups. Logistic regression analysis was performed to identify independent risk factors associated with poor prognosis. There were no statistically significant differences in baseline characteristics between the 2 groups, indicating good comparability. The proportion of laparoscopic surgeries was higher in the good prognosis group, and both intraoperative blood loss and operative time were significantly lower compared to the poor prognosis group (*P* < .05). The incidence of postoperative complications was significantly higher in the poor prognosis group (40.6% vs 19.1%, *P* = .006), along with prolonged hospital stay and delayed functional recovery (*P* < .05). One-year recurrence and overall survival rates also differed significantly between the groups (recurrence: 32.8% vs 10.3%, *P* = .002; survival: 71.9% vs 91.2%, *P* = .004). Multivariate logistic regression analysis identified intraoperative blood loss > 500 mL (OR = 2.36, *P* = .019) and postoperative complications (OR = 3.12, *P* = .003) as independent predictors of poor prognosis. The postoperative prognosis of elderly patients with gynecologic cancers is influenced by multiple factors, among which intraoperative bleeding control and complication management are critical intervention points. Preoperative risk assessment, surgical optimization, enhanced perioperative care, and structured postoperative rehabilitation should be emphasized to improve recovery quality and long-term survival in this high-risk population.

## 1. Introduction

With the accelerating trend of population aging, the incidence of gynecologic malignancies among elderly women has been increasing year by year, posing a significant threat to their quality of life and life expectancy.^[1,2]^ According to data from the National Cancer Center of China, women aged 65 years and older account for a growing proportion of cases in common gynecologic malignancies such as endometrial cancer, ovarian cancer, and cervical cancer.^[3–5]^ Elderly patients with gynecologic cancers often exhibit age-related physiological decline, compromised immune function, and multiple chronic comorbidities, placing them at greater risk when undergoing aggressive treatments such as surgery.^[6]^ While surgical intervention remains a cornerstone of treatment for most gynecologic malignancies, elderly patients are prone to higher rates of postoperative complications, prolonged recovery times, and poorer long-term survival, which can significantly reduce the overall therapeutic benefit.^[7–10]^ Consequently, accurate assessment of surgical suitability, optimization of perioperative management, and identification of prognostic factors in elderly patients have become key focal points in clinical research.

In recent years, the introduction of minimally invasive surgery, enhanced recovery after surgery protocols, and comprehensive geriatric assessment has opened new opportunities for surgical treatment in older populations. Studies have demonstrated that laparoscopic and other minimally invasive techniques can reduce surgical trauma and postoperative complications without increasing operative time or risk. Additionally, multidisciplinary perioperative care models have gained traction, integrating nutritional support, preoperative risk assessment, and rehabilitation strategies into the surgical preparation process for elderly patients.^[11–13]^ However, most existing studies have focused on a single cancer type or isolated perioperative factors, lacking a comprehensive analysis that correlates postoperative recovery quality with mid- to long-term survival outcomes in elderly patients undergoing gynecologic cancer surgery.

Furthermore, traditional prognostic studies tend to emphasize tumor-related characteristics such as stage, histological subtype, and treatment modality. However, in elderly patients, outcomes are often influenced by a combination of intraoperative risk factors, postoperative complications, and recovery speed – factors that are especially pertinent in this population.^[14–16]^ Therefore, a comprehensive evaluation encompassing intraoperative events, postoperative recovery, and follow-up outcomes is essential for accurately assessing the effectiveness of surgical treatment in elderly women with gynecologic malignancies. Such an approach could inform individualized treatment planning and support the development of tailored prognostic assessment systems for geriatric patients. In the context of precision medicine and geriatric care integration, there is an urgent need for more comprehensive studies to develop safer, more effective, and feasible treatment strategies for this growing patient population.

Based on this background, the present study retrospectively analyzed perioperative features, postoperative recovery, and 1-year prognostic outcomes in 132 patients who underwent surgical treatment for gynecologic cancers at our institution between January 2023 and May 2024. The objective was to identify key factors affecting postoperative recovery quality and long-term outcomes, and to explore associations among minimally invasive surgery, perioperative management, and recovery trajectories.

## 2. Materials and Methods

### 2.1. Study population and grouping

This study was approved by the Ethics Committee of the First Affiliated Hospital of Xiamen University on January 15, 2023. This was a retrospective cohort study that included 132 elderly patients with gynecologic malignancies who underwent surgical treatment at our institution between January 2023 and May 2024. All patients were aged 65 years or older. The types of gynecologic malignancies included endometrial cancer, ovarian cancer, cervical cancer, and fallopian tube cancer. All diagnoses were confirmed by histopathological examination, and all cases were primary malignant solid tumors.

Inclusion criteria:

1. Patients with histologically confirmed primary gynecologic malignancies;
2. Age ≥ 65 years;
3. Underwent complete surgical resection with no evidence of distant metastasis preoperatively;
4. Completed at least 1 year of standardized postoperative follow-up with complete follow-up data;
5. Availability of comprehensive preoperative clinical data including demographic information, perioperative parameters, and postoperative recovery indicators.

Exclusion criteria:

1. History of other malignant tumors or multiple primary malignancies;
2. Perioperative mortality or loss to follow-up due to severe postoperative complications;
3. Presence of severe organ dysfunction (e.g., end-stage heart, lung, or renal failure) that may affect prognostic evaluation;
4. Received palliative care postoperatively;
5. Incomplete follow-up or missing follow-up records.

### 2.2. Grouping method

Patients were categorized into either a good prognosis group or a poor prognosis group based on their postoperative recovery quality and 1-year follow-up outcomes. Specifically, patients with smooth postoperative recovery, absence of severe complications, and no evidence of recurrence within 1 year were assigned to the good prognosis group (n = 68). Patients who experienced delayed recovery, severe complications, tumor recurrence, or death within 1 year were classified into the poor prognosis group (n = 64). This classification integrates both short-term recovery quality and mid-term oncologic outcomes, providing a more comprehensive assessment of the patients’ overall prognosis after surgery.

### 2.3. Baseline characteristics and perioperative parameters

Data collected included demographic information (age, height, weight, body mass index [BMI]), vital signs (systolic and diastolic blood pressure, heart rate), and medical history (gravidity, smoking status, and comorbidities). Tumor-related features included FIGO stage, histologic type, and tumor size. Perioperative technical indicators included surgical approach (laparoscopic vs open surgery), intraoperative blood loss, and operative time.

### 2.4. Postoperative recovery and follow-up assessment

Perioperative recovery indicators included length of hospital stay, time to first ambulation, and time to gastrointestinal function recovery. Postoperative complications (e.g., infections, bleeding, urinary retention, and bowel obstruction) were recorded according to standard clinical diagnostic criteria. All patients underwent a 1-year follow-up period to assess tumor recurrence and overall survival status.

### 2.5. Statistical analysis

All data were analyzed using SPSS version 26.0 (IBM Corp., Armonk). Continuous variables were expressed as mean ± standard deviation and compared between groups using independent-samples *t* tests. Categorical variables were presented as counts and percentages, and compared using the chi-square (*χ*²) test. A 2-sided *P* value of < .05 was considered statistically significant.

### 2.6. Logistic regression modeling

To identify independent risk factors associated with poor postoperative prognosis, univariate logistic regression analyses were initially performed. Variables with statistical significance (*P* < .05) were subsequently included in a multivariate logistic regression model. Odds ratios and 95% confidence intervals (CI) were calculated to determine independent predictors of adverse outcomes following surgery.

## 3. Result

### 3.1. Comparison of baseline characteristics

A total of 132 elderly patients (aged ≥ 65 years) with gynecologic cancers who underwent surgical treatment at our hospital between January 2023 and May 2024 were included in this study. The mean age was 72.4 ± 5.1 years. Based on postoperative recovery status and 1-year follow-up outcomes, patients were divided into a good prognosis group (n = 68) and a poor prognosis group (n = 64). A comparison of demographic characteristics, physiological parameters, tumor-related features, and comorbidities between the 2 groups revealed no statistically significant differences in age, height, weight, BMI, systolic and diastolic blood pressure, heart rate, gravidity, smoking history, hypertension, diabetes, FIGO stage, tumor histological type, or tumor size (all *P* > .05). These findings indicate that the 2 groups were well matched at baseline, thus providing a reliable foundation for subsequent analyses. Details are presented in Table 1.

**Table 1:** Comparison of baseline characteristics between the 2 groups of elderly gynecologic cancer patients.

Variable	Good prognosis group (n = 68)	Poor prognosis group (n = 64)	*χ*²/*t* value	*P* value
Age (yr)	72.1 ± 5.0	72.7 ± 5.2	0.659	.511
Height (cm)	157.3 ± 6.4	158.0 ± 6.0	0.645	.52
Weight (kg)	58.2 ± 8.7	59.0 ± 9.2	0.525	.601
BMI (kg/m²)	23.5 ± 2.9	23.1 ± 3.2	0.738	.462
Systolic blood pressure (mm Hg)	138.7 ± 12.5	140.2 ± 13.1	0.672	.503
Diastolic blood pressure (mm Hg)	81.4 ± 9.3	82.1 ± 8.9	0.458	.648
Heart rate (beats/min)	76.3 ± 7.1	77.0 ± 6.8	0.572	.568
Smoking history (n, %)	8 (11.8%)	10 (15.6%)	0.389	.533
Gravidity ≥ 1 (n, %)	59 (86.8%)	53 (82.8%)	0.432	.511
Hypertension (n, %)	31 (45.6%)	33 (51.6%)	0.505	.477
Diabetes mellitus (n, %)	18 (26.5%)	20 (31.3%)	0.369	.544
FIGO stage (I/II/III+)	24/22/22	20/19/25	0.859	.651
Tumor histology (adeno/squamous/others)	38/20/10	34/18/12	0.514	.773
Tumor size (cm)	4.2 ± 1.3	4.4 ± 1.5	0.805	.422

BMI = body mass index.

### 3.2. Comparison of surgical approach and intraoperative parameters

**Figure 1.**
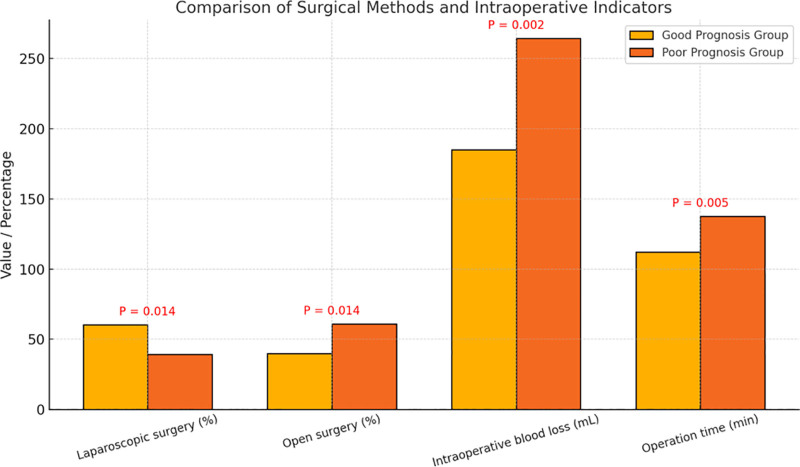
Comparison of surgical methods and intraoperative indicators.

As shown in Figure 1, a significantly higher proportion of patients in the good prognosis group underwent laparoscopic surgery compared to the poor prognosis group (60.3% vs 39.1%, *P* = .014), while the proportion of open surgeries was higher in the poor prognosis group (60.9% vs 39.7%, *P* = .014). These findings suggest that minimally invasive surgery may contribute to improved postoperative recovery and long-term outcomes. Regarding intraoperative parameters, the poor prognosis group experienced significantly greater intraoperative blood loss compared to the good prognosis group (264.5 ± 76.8 mL vs 185.2 ± 64.3 mL, *P* = .002), as well as longer operative time (137.6 ± 25.4 minutes vs 112.3 ± 21.8 minutes, *P* = .005). All differences were statistically significant.

### 3.3. Comparison of postoperative complication rates

**Figure 2.**
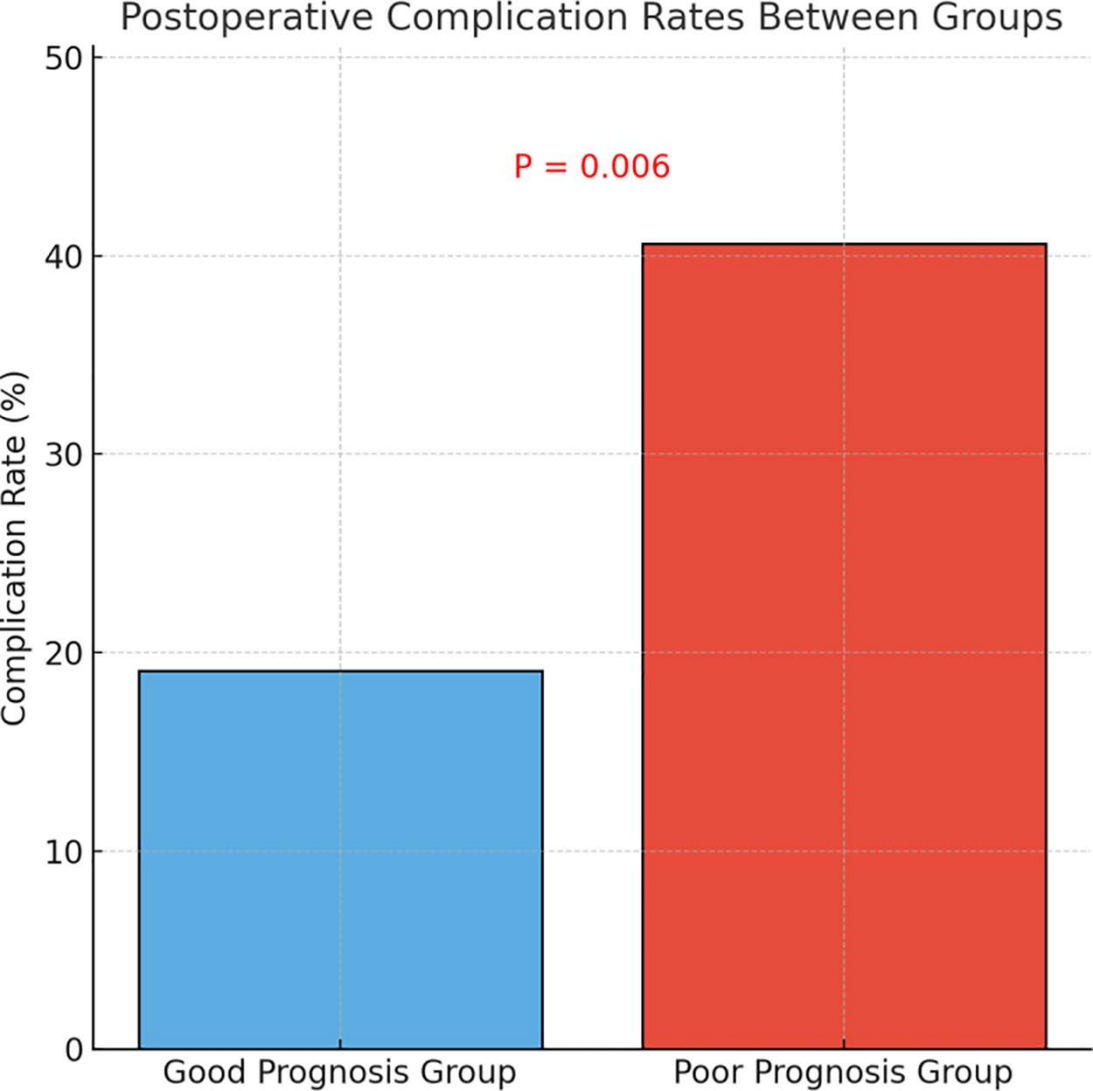
Postoperative complication rates between groups.

This study further analyzed the distribution of postoperative complications – including postoperative infection, hemorrhage, urinary retention, and intestinal obstruction – among patients with different prognostic outcomes. As shown in Figure 2, the incidence of postoperative complications was significantly higher in the poor prognosis group (40.6%) compared to the good prognosis group (19.1%), with the difference being statistically significant (*P* = .006). These findings suggest that effective control of postoperative complications plays a critical role in improving recovery quality and long-term outcomes. Therefore, enhanced intraoperative and perioperative risk management and early intervention are essential for elderly patients with gynecologic cancers.

### 3.4. Length of hospital stay and postoperative recovery

**Figure 3.**
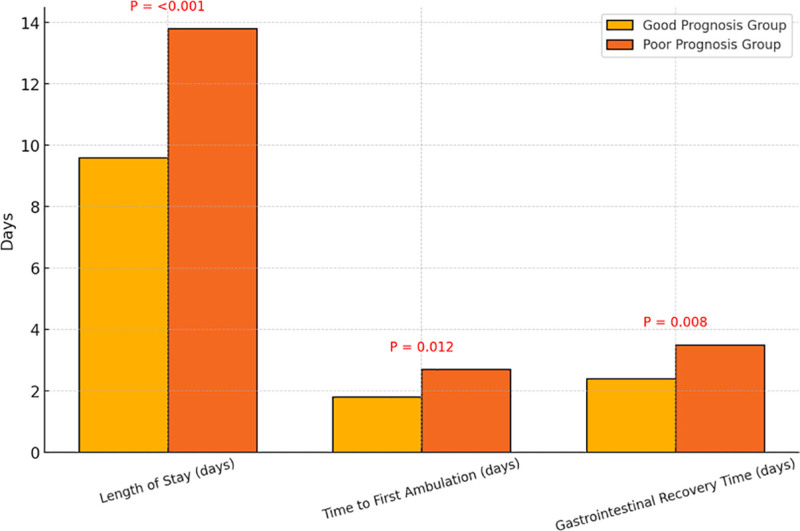
Comparison of postoperative recovery indicators between groups.

Postoperative recovery indicators revealed a significantly delayed recovery process in the poor prognosis group. As illustrated in Figure 3, the average length of hospital stay was significantly longer in the poor prognosis group (13.8 ± 3.4 days) compared to the good prognosis group (9.6 ± 2.8 days, *P* < .001). Similarly, the time to first ambulation after surgery was delayed in the poor prognosis group (2.7 days vs 1.8 days, *P* = .012), and the time to gastrointestinal function recovery was also extended (3.5 days vs 2.4 days, *P* = .008). These findings suggest a close relationship between the speed of early postoperative recovery and overall prognosis. They highlight the importance of optimizing perioperative care protocols and promoting early functional rehabilitation to improve both short-term recovery and long-term outcomes in elderly gynecologic cancer patients.

### 3.5. Comparison of recurrence and overall survival rates during follow-up

**Figure 4.**
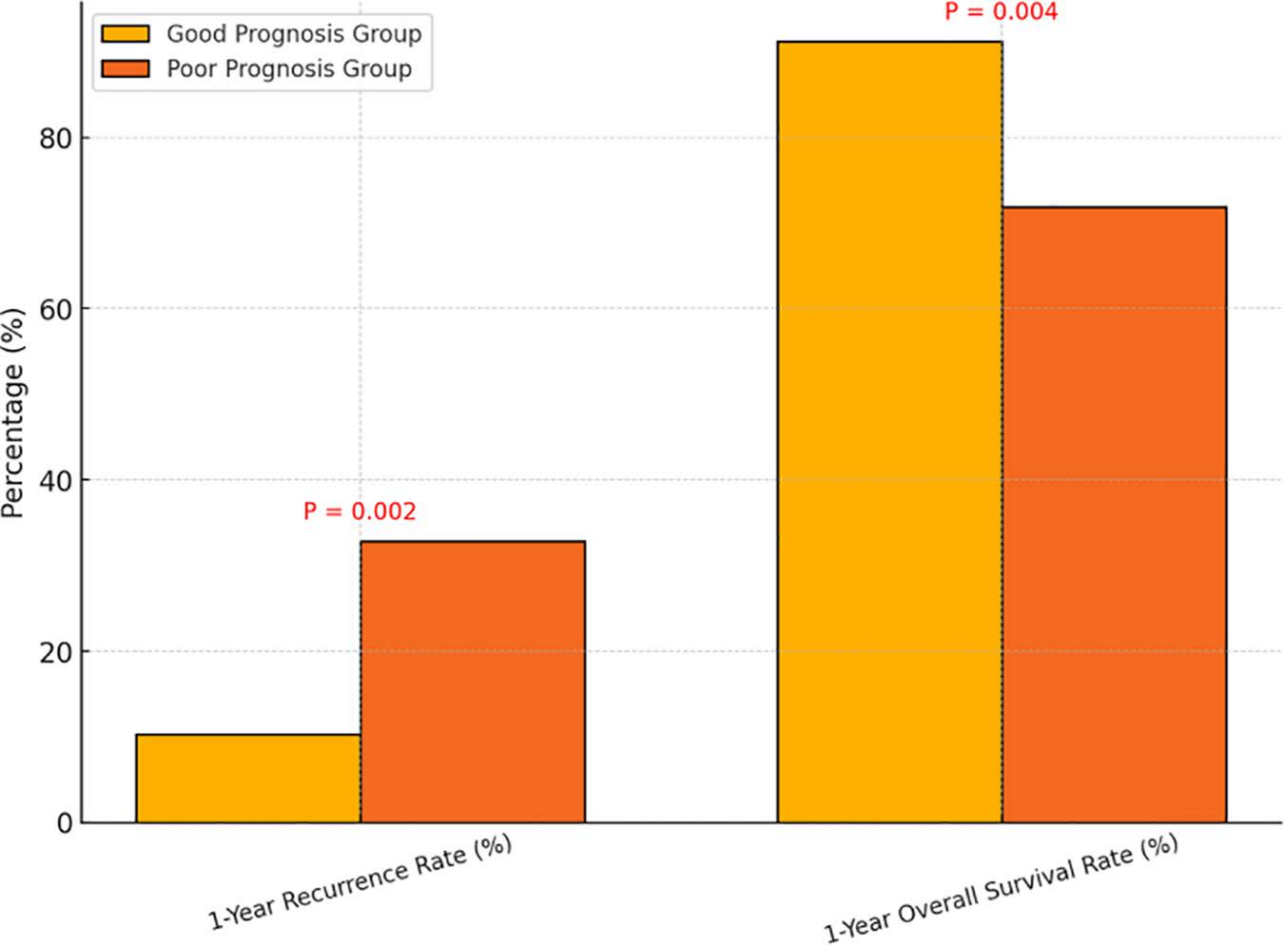
Comparison of 1-year recurrence and overall survival rates between prognostic groups.

Follow-up data up to May 2025 demonstrated significant differences in 1-year recurrence and overall survival rates between the good and poor prognosis groups. The 1-year recurrence rate was markedly higher in the poor prognosis group (32.8%) compared to the good prognosis group (10.3%, *P* = .002). Similarly, the 1-year overall survival rate was significantly lower in the poor prognosis group (71.9%) than in the good prognosis group (91.2%, *P* = .004), as shown in Figure 4. These findings indicate that early postoperative recovery status not only affects the incidence of short-term complications but is also closely associated with mid- to long-term risks of tumor recurrence and overall survival outcomes. This underscores the critical importance of optimizing perioperative management to improve long-term prognosis in elderly patients with gynecologic cancers.

### 3.6. Univariate and multivariate logistic regression analysis

To identify the factors associated with poor postoperative prognosis in elderly patients with gynecologic cancers, logistic regression analysis was performed on perioperative clinical variables. Univariate analysis revealed that surgical approach (laparoscopy vs laparotomy), intraoperative blood loss, postoperative complications, and length of hospital stay were significantly associated with prognosis (*P* < .05), suggesting their potential role as risk factors.

Subsequently, these variables were included in a multivariate logistic regression model. The analysis demonstrated that intraoperative blood loss > 500 mL (OR = 2.36, 95% CI: 1.15–4.86, *P* = .019) and postoperative complications (OR = 3.12, 95% CI: 1.47–6.64, *P* = .003) were independent risk factors for poor prognosis. In contrast, surgical approach and hospital stay did not remain statistically significant in the multivariate model (*P* > .05), indicating that their independent impact on prognosis was limited after adjusting for other variables.

These findings highlight the importance of intraoperative hemostasis and complication prevention in improving postoperative outcomes among elderly patients undergoing surgery for gynecologic malignancies. Detailed results are presented in Table 2.

**Table 2:** Results of univariate and multivariate logistic regression analyses.

Variable	Univariate *P* value	Multivariate OR	95% confidence interval	Multivariate *P* value
Surgical approach (laparoscopy vs open)	.021	–	–	>.05
Intraoperative blood loss > 500 mL	.008	2.36	1.15–4.86	.019
Postoperative complications (yes vs no)	.002	3.12	1.47–6.64	.003
Length of hospital stay (per d)	.015	–	–	>.05

OR = odds ratios.

## 4. Discussion

Elderly patients with gynecologic malignancies have long been considered a high-risk group for surgical treatment due to reduced physiological reserve, a high burden of comorbidities, and poor postoperative recovery capacity.^[17,18]^ With the increasing aging of the population in China, the incidence of gynecologic cancers in elderly women has been rising annually. How to scientifically evaluate perioperative risk and optimize postoperative recovery pathways in this population has become a key focus in clinical practice.^[19]^ This study analyzed elderly gynecologic cancer patients who underwent surgery at our center over the past 2 years, aiming to identify factors associated with postoperative recovery and 1-year prognosis to inform individualized treatment strategies.

In the baseline comparison, no significant differences were found between the good and poor prognosis groups in age, body weight, BMI, vital signs, tumor stage, or histologic type, suggesting good overall comparability and providing a sound foundation for subsequent analysis. Regarding surgical approach, the proportion of laparoscopic procedures was significantly higher in the good prognosis group, while the poor prognosis group had a higher rate of open surgeries, indicating that minimally invasive techniques may offer advantages in postoperative recovery. Similar findings have been reported in previous studies of elderly patients with endometrial cancer, where laparoscopy was associated with reduced intraoperative blood loss, shorter hospital stays, and fewer postoperative complications.^[20]^

Intraoperative parameters also differed significantly between groups. Patients in the poor prognosis group experienced more blood loss and longer operative times. Excessive intraoperative bleeding not only increases the burden of postoperative recovery but may also impair immune function and delay physiological reconstruction. Prior studies have identified intraoperative blood loss as a key predictor of perioperative complications in elderly patients, consistent with our findings.^[21]^

In terms of postoperative complications, the poor prognosis group had a significantly higher incidence, primarily including infections, urinary retention, and bowel obstruction. These complications directly impact recovery quality and length of hospital stay. Postoperative complications have also been linked to reduced survival in elderly patients. A study on ovarian cancer, for example, found that early postoperative complications significantly increased tumor recurrence and mortality rates.^[22]^

Recovery speed is also crucial for long-term outcomes. In this study, the poor prognosis group had significantly prolonged hospital stays, delayed ambulation, and slower gastrointestinal function recovery, indicating an overall delayed postoperative functional recovery. Previous studies suggest that early postoperative functional recovery is associated with improved immune status, better quality of life, and more favorable long-term tumor control. Therefore, optimizing postoperative care and encouraging early functional rehabilitation are essential strategies.

With respect to survival outcomes, the good prognosis group had significantly lower 1-year recurrence rates and higher survival rates compared to the poor prognosis group. Kaplan–Meier survival analysis and follow-up data indicate that early recovery not only improves short-term complication control but also positively influences medium- to long-term tumor control.

Logistic regression further identified independent predictors of poor prognosis. Intraoperative blood loss >500 mL and the presence of postoperative complications were significantly associated with adverse outcomes. These findings underscore the critical importance of managing intraoperative blood loss and preventing complications during the perioperative period. Similar conclusions have been reported in studies of prognostic factors in elderly cervical cancer patients.^[23]^

A key innovation of this study lies in the integrated consideration of intraoperative variables, postoperative recovery, and long-term prognosis. We adopted a composite endpoint combining “postoperative recovery quality + 1-year recurrence/survival” to classify prognostic outcomes, providing a more clinically relevant stratification. Furthermore, we employed multivariate models to systematically identify modifiable independent risk factors, offering data support for precision surgical management.

Despite the valuable insights from this retrospective, single-center study, there are several limitations. One key limitation is the potential for unmeasured confounders, such as American Society of Anesthesiologists physical status scores, frailty indices, and nutritional status, which were not systematically assessed. These factors are particularly relevant in geriatric populations and can independently affect surgical outcomes, recovery, and survival. The absence of these variables limits our ability to fully account for their impact on our findings. Future studies should incorporate these factors through prospective designs or comprehensive geriatric assessments to better understand their role in postoperative outcomes for elderly patients with gynecologic malignancies. Additionally, patients who were lost to follow-up or who died early postoperatively (within 30 days) were excluded from the analysis to avoid selection bias. While this exclusion may underrepresent sicker patients and limit the generalizability of our findings, it was necessary to ensure the integrity of our analysis of postoperative recovery and long-term outcomes. Future research should consider including a broader cohort to assess the impact of early mortality and incomplete follow-up on surgical outcomes.

In conclusion, postoperative prognosis in elderly patients with gynecologic cancers is influenced by multiple factors, among which intraoperative blood loss control and complication management are pivotal. Early recovery quality is closely linked to long-term outcomes. Emphasis should be placed on preoperative risk assessment, surgical approach optimization, and enhanced perioperative care to improve recovery quality and survival in this vulnerable population.

## 5. Conclusion

This study focused on evaluating postoperative recovery and its impact on long-term outcomes in elderly patients with gynecologic malignancies. Our analysis identified intraoperative blood loss and postoperative complications as independent risk factors for adverse postoperative outcomes. Notably, favorable postoperative recovery was associated with a reduced incidence of complications, a lower 1-year recurrence rate, and improved overall survival. For the first time, we incorporated a composite endpoint – postoperative recovery quality combined with 1-year survival status – to stratify prognosis. This multidimensional approach offers a more comprehensive reflection of patient outcomes and provides a novel perspective for preoperative risk assessment and perioperative management. These findings underscore the importance of enhancing intraoperative risk control, optimizing postoperative care pathways, and implementing early functional rehabilitation strategies to improve treatment efficacy and long-term survival quality in elderly patients with gynecologic cancers.

## Author contributions

**Conceptualization:** Yuanqing Chen, Zhengyi Chen.

**Data curation:** Yuanqing Chen, Zhengyi Chen.

**Formal analysis:** Yuanqing Chen, Zhengyi Chen.

**Funding acquisition:** Yuanqing Chen.

**Investigation:** Yuanqing Chen, Zhengyi Chen.

**Methodology:** Yuanqing Chen, Zhengyi Chen.

**Supervision:** Yuanqing Chen, Zhengyi Chen.

**Validation:** Zhengyi Chen.

**Visualization:** Yuanqing Chen.

**Writing – original draft:** Yuanqing Chen, Zhengyi Chen.

**Writing – review & editing:** Yuanqing Chen, Zhengyi Chen.
